# Researching Healthy Public Policy: Navigating the ‘Black Box’ Means Thinking More About Power: Comment on "Developing a Framework for a Program Theory-Based Approach to Evaluating Policy Processes and Outcomes: Health in All Policies in South Australia"

**DOI:** 10.15171/ijhpm.2018.52

**Published:** 2018-06-03

**Authors:** Patrick Harris

**Affiliations:** University of Sydney, School of Public Health, Menzies Centre for Health Policy, Sydney, NSW, Australia.

**Keywords:** Health, Public Policy, Logic Models, Power

## Abstract

Lawless et al provide a valuable narrative of using program logic to develop an evaluation of Health in All Policies (HiAP) in South Australia. In this commentary I argue that the paper and analysis is an extremely useful example of navigating the supposed black box of policy-making. However the original makes the reader work too hard and is distracting from the main narrative of explaining the logic behind the HiAP approach in South Australia. My response covers avoiding epistemological traps and weighing up the pragmatics of collaborative policy research with more complex institutional policy issues like power

The original piece of research by Lawless et al^1^ is exemplary in its aim and description of using program logic to improve understandings of the practice of Health in All Policies (HiAP) in South Australia. Its main weakness is that it contains too many concepts and is, to use an English expression, ‘Too clever by half.’ A kaleidoscope of ideas are presented, with new ideas coming in across the whole paper making the whole thing very hard work for the reader. That said, the paper does provide important knowledge that has been missing in the healthy public policy arena (I prefer the term Healthy Public Policy to HiAP as like Lawless et al I view the former as the discipline and the latter an approach).

Taking a global perspective, the Lawless et al original contributes to an increasingly important body of work about influencing the wider determinants of health through healthy public policy. It has been 10 years since the World Health Organization (WHO) Commission on the Social Determinants of Health provided a global evidence base of health inequities and their causes.^2^ That evidence however largely eschewed any engagement with politics and political science.^3^ There are now groups of researchers internationally who are explicitly using political science to address the problem of getting health into public policy. Fran Baum and the team behind the Lawless et al paper are leading the Australian charge in this endeavour, especially through our (disclaimer – I am involved) NHMRC Centre for Research Excellence.^4^ Each of us also has our own focus areas, mine being infrastructure and urban planning.^5,6^ Baum’s team have been inextricably involved in the HiAP endeavours in South Australia, as well as progressing program logic as an approach for policy analysis, both of which form the Lawless article.

My first critique concerns the research being unable to take on outcome evaluation and providing a lengthy and detailed explanation of this. Unlike the response to the article by Labonte^7^ (who rather confusingly critiques the lack of outcome evaluation but then details why this was the case), I think the paper would be much improved by being more direct and succinct about this. I would prefer the original (p. 2 does this) to simply detail the program theory approach as a process, mixing theory and practical experience within a theory based evaluation paradigm, to articulate the logic between program activities and their ‘presumed’ potential outcomes. All the other clever concepts about causal relationships, contribution vs. attribution etc then become moot and removing them would leave the paper less ‘noisy.’ Yes, methodological depth is required for research and evaluation, and there is a real issue going on here about different scientific approaches to causation. This type of article is not a PhD thesis requiring this sort of depth. Rather than being distracted from the narrative by detailed – and well-worn – epistemology I’d rather see judicious referencing.

Relatedly, the strength in the paper is the narrative developed to explain the process of developing the program logic framework ‘that has guided the evaluation of the South Australian HIAP initiative’ (p. 3). This success has several pragmatic and conceptual dimensions. Pragmatically the involvement of policy-makers is outstanding. This involvement is no mean feat and indeed may be a bi-product of the relationships developed as part of the HiAP approach in South Australia. Active collaboration and deliberation with policy-makers is very difficult to achieve. It takes entrepreneurialism, time, tenacity, relationships, and above all an institutional mandate to make initiatives such as HiAP to make it work. There is great merit in bringing policy-makers to articulate the dimensions of the logic model, using theories of the policy process to trigger thinking and explanation. We know that policy learning is the principle mechanism for policy change,^8-10^ and understanding program logic as a way of working to achieve this is very useful indeed. Explaining the actual doing of this adds enormously to the literature and helps add program logic as another useful health and public policy process. That said, it is worth noting that program theory-based evaluation is not, as the paper claims, ‘currently the best approach’ (p. 1 – ‘implications for policy-makers’) for prospective healthy public policy work. Other equally useful approaches include health impact assessment, complex systems analysis, and adaptive management – although the application of these could certainly do with the same detailed policy analysis as that provided by Lawless et al.

Conceptually the articulation of the policy dimensions that go into the framework and the analysis supports much of the policy literature and is another strength for the field of healthy public policy. The analysis is based around the core dimensions known to make up policy institutions (or sub-systems) in political science: Actors, structures, ideas leading to policy choices.^8,11^ In the analysis here, these core dimensions act as the institutional glue that makes the analysis work. I use these dimensions at the core of my own work and cannot stress enough how important these are for unpacking the supposed ‘Black box’ of policy-making.^12^ One of the notoriously difficult things to do in theory based policy evaluation is to articulate the mechanisms at play in policy-making, and these concepts allow a stratified approach to that analysis (where each concept overlaps with but is analytically distinct from the other). However, just as Lawless et al present in their framework (Figure 1), it is crucial to realise that these essential dimensions are not the full story, instead they provide the platform for a fuller narrative to unfurl.

Where the analysis in the original struggles is shifting that story up a ‘critical’ gear to include a stronger articulation of the institutional context. Crucial issues such as power and politics end up only nodded too (noting that power was the subject of other recent commentaries on the original). Lawless et al do introduce power (p. 6) as part of implementation of strategies (power trumps evidence), but do not elaborate much beyond this in terms of the structural norms and mandates that flow through to influence policy choices. One passage is particularly interesting when seen with this type of critical lens.

“*The ultimate goal of the HiAP intervention was the subject of considerable debate within and following the workshops. Early drafts of the framework posited ‘increased population health and health equity’ as the ultimate goal. A number of workshop participants suggested this goal did not reflect non-health sectors’ objectives or the aim of achieving co-benefits. The final version of the framework incorporates concerns larger than health, phrased as: ‘SA Government’s goal of making SA a better place to live with increased population health and equity’”* (pp. 6-7).

Having to re-frame to be the ‘SA Government’s goal’ here was in all likelihood well-meaning. Behind this however, is that the HiAP approach in SA is internally government focussed. This could have and should be questioned in terms of who holds what power over who, why, and ultimately with what effect? Who, for instance, is actually represented by ‘The SA Government’? Non-governmental organizations (NGOs) and community groups would be unlikely to want the government to hold such power over them by dictating their goals. Extending this is the crucial place of ‘governance’ in policy-making, where powerful influences on policy come from outside of government such as NGOs, community groups and corporate stakeholders. Governance however is given a wide berth by Lawless et al beyond being an internal mechanism for intra-agency engagement.

But this type of analysis is easier to critique than it is to do. On the one hand this is constrained by working collaboratively with policy-makers who might not always recognise, or be willing to discuss, the power structures inherent their work. On the other, power is a tricky and slippery concept. My own experience is that power in policy becomes clearest when analysis brings in differences in the systems that different stakeholders in the policy inhabit and the values that these systems enable or constrain: communities, for instance, view policy through its impact on their lifeworld whereas policy-makers work for administrative or broader goals (such as economic rationalism, etc).^7^

Concerning this type of institutional, power laden, analysis, another analytic question is how far to take it? Labonte’s response to the original makes interesting observations about linking HiAP to global forces, but again this line of argument is easier to make than to address. The end point of this type of analysis inevitably ends up with a critique of Capital(ism) and/or Neoliberal nefariousness. This is all well and good but such analysis risks being disempowering. However, if we take it that healthy public policy is ultimately about challenging normative positions about policy choices such that policies improve rather than imperil health, then this brings agency, and a positive use of power, into the mix. This nevertheless becomes explicitly political, as it should, bringing in a deeper understanding of institutions and their influence on policy decisions and, eventually, outcomes.

The Lawless article suggests this type of analysis was either not useful for HiAP policy partners and/or not put on the table. This probably had a pragmatic component to it. Unpacking structural dynamics (where structures are defined as the rules and mandates that flow through systems) are necessary but insufficient in the absence of any type of strategies for change. Particularly where equity is central goal for a policy endeavour like HiAP, the challenge for program logic type approaches is to articulate how policies or programs can at least recognise these wider influences. To reiterate, this does not have to be all doom and gloom – power can be used for positive change just as it can dominate and exploit. I enjoyed de Leeuw’s^13^ response to the Lawless et al piece concerning power and suggest others either doing or analysing HiAP type initiatives think in the manner she suggests, including channelling Che Guevara through Beyonce Knowles.

In conclusion, the Lawless article provides a worthwhile and important analysis of a complex policy initiative using program logic. The epistemological distractions might well be because the authors tried to take on the outcome measurement obsession in Public Health. The fact is that program logic works by articulating how policy processes lead to presumed outcomes. That said, the original adds greatly to the body of literature that is helping to articulate navigating the black box of policy-making to create healthy public policy. As articulated here and in the other responses to the original, bringing power explicitly into analysing healthy public policy is the next challenge facing the field.

## Ethical issues

Not applicable.

## Competing interests

Author declares that he has no competing interests.

## Author’s contribution

PH is the single author of the paper.

## Funding

The author is funded by the Australian National Health and Medical Research Council APP1090644
